# User evaluation of AI- and human-generated responses in digital health communication: a paired survey study

**DOI:** 10.1186/s12913-026-15305-4

**Published:** 2026-08-19

**Authors:** Christin Schulz, Patrycja Adämmer, Emma Fechner, Eva Kassberg, Ida Laux, Ella Pollatscheck, Katja Porsch, Lady Romamti, Iryna Tymchuk, Johannes Gräske

**Affiliations:** Department II: Health, Education, and Training, Alice Salomon Hochschule Berlin, Alice-Salomon-Platz 5, 12627 Berlin, Germany

**Keywords:** Artificial intelligence, Digital health communication, Patient perspective, Health information, Empathy, Health services research

## Abstract

**Background:**

Digital health communication is gaining importance as health care systems face increasing demand and structural constraints. Generative artificial intelligence (AI) tools such as ChatGPT are increasingly used for health information seeking; however, direct comparisons between AI-generated and human responses from the user perspective remain limited.

**Methods:**

A quantitative exploratory cross-sectional online survey using a paired-response design was conducted in Germany. The survey instrument was developed based on the study objectives and refined through pretesting. Participants evaluated two responses to the same real-world health query: one written by a physiotherapist and one generated by ChatGPT-4. Outcomes included perceived empathy, comprehensibility, perceived discriminatory content in the responses, and expert-rated clinical quality. Paired comparisons were analyzed using Wilcoxon signed-rank tests, and logistic regression models were applied to explore associations between participant characteristics and preference patterns.

**Results:**

The analytical sample comprised 224 participants. Across most items, the AI-generated response was rated more favorably than the human response, particularly with regard to comprehensibility and empathy (all *p* < .001). The AI-generated response was rated more favorably across both empathy and comprehensibility items, with highly comparable preference distributions between the two domains. Perceptions of discriminatory content were rare and did not differ significantly between responses. Expert evaluations also favored the AI-generated response in terms of clinical accuracy and completeness, although these findings should be interpreted cautiously given the exploratory design.

**Conclusions:**

The findings suggest that participants perceived the AI-generated response as aligning more closely with central user expectations for text-based health information, particularly regarding clarity and supportive language, although these findings should be interpreted cautiously given the substantial differences in length and structure between responses and the exploratory single-case design. At the same time, these perceptions should be considered alongside ongoing concerns regarding the clinical appropriateness, safety, and equity of AI-generated health information, which were not comprehensively assessed in the present study. The present findings therefore primarily provide evidence regarding user-perceived communication quality rather than the clinical validity of AI-generated responses. Accordingly, further research is needed to establish the clinical appropriateness, safety, guideline concordance, and potential role of AI-generated responses in healthcare consultation.

**Supplementary Information:**

The online version contains supplementary material available at 10.1186/s12913-026-15305-4.

## Background

### Digital health communication under increasing pressure

Access to timely and reliable health advice is becoming more challenging across many health care systems. Workforce shortages, demographic change, and rising demand for care are contributing to structural constraints that affect both the availability and quality of professional consultation. At the same time, health communication is increasingly shifting into digital environments. In the European Union, 93% of individuals aged 16–74 years reported using the internet in 2024 [1], and among internet users, 63% searched online for health-related information such as illness, nutrition, or health promotion [2].

While digital access may expand the reach of health information, it also raises important questions about the quality of communication—particularly with respect to comprehensibility, structural clarity, empathy, and the potential for biased or discriminatory content.

### Expansion of generative AI in health information seeking

Beyond traditional digital sources such as search engines, online portals, and forums, generative artificial intelligence (AI) systems are becoming increasingly prominent in the context of health information seeking. Tools such as ChatGPT provide immediate, anonymous, and interactive responses, which may lower barriers to accessing advice. International data indicate that the use of ChatGPT for health-related questions is already widespread in the general population and varies across sociodemographic groups [3].

### Context-dependent acceptance of digital counseling

Acceptance of digital consultation is not uniform but appears to depend strongly on situational factors. Virtual formats are often perceived as appropriate for clearly defined concerns, whereas direct interpersonal communication tends to be preferred in cases involving initial contact, greater uncertainty, or complex health situations [4, 5]. At the same time, qualitative research points to unintended consequences of digital consultations, including communication difficulties and the risk of disadvantaging digitally excluded populations [6].

The nature of the health concern itself also appears to be relevant. Chatbots may be viewed more favorably when addressing stigmatized topics, yet skepticism tends to increase when symptoms are perceived as serious [7].

### Perceived communication quality of AI-generated responses

Emerging evidence suggests that AI-generated responses may be evaluated positively with regard to core communication characteristics. A systematic review with meta-analysis reported that in text-based study settings, AI chatbots sometimes received higher empathy ratings than human health care professionals, although methodological limitations should be taken into account [8]. Analyses based on real-world health forum posts similarly found chatbot responses to be more frequently rated as empathetic and higher in overall quality than physician responses [9]. At the same time, domain-specific evaluations indicate that ChatGPT can explain complex information in an accessible manner but requires systematic scrutiny with regard to accuracy [10].

### Clinical limitations, safety concerns, and bias

Despite promising evaluations of communication quality, several studies emphasize limitations in the clinical appropriateness of AI-generated advice, particularly for complex medical questions in which human expertise often remains superior [11]. Concerns have also been raised about inconsistent response quality and the possibility of harmful recommendations [12]. In addition, issues of fairness and equity warrant attention. Tools for systematically identifying bias and potential equity harms in large language model (LLM) outputs have been proposed, underscoring the relevance of discrimination-sensitive evaluation [13].

### Research gap and study objective

Although a growing body of literature examines AI-supported health communication, direct comparisons between AI-generated and human responses remain limited - particularly when both are based on the same real-world user query and evaluated across multiple communication dimensions from the user perspective. Empathy, comprehensibility, and non-discrimination represent central dimensions of healthcare communication, linked to patient satisfaction, trust, and the ability to act on health information [14–19]. The objective of this study was to empirically compare a ChatGPT-4 response with the response of a physiotherapist to a real question from an online health forum. By examining user evaluations across key communication dimensions, the study aims to contribute to a more differentiated understanding of the potential, limitations, and risks of AI-supported digital health communication in comparison with human consultation.

### Methods

A quantitative exploratory cross-sectional online survey using a paired-response evaluation design was conducted. Each participant evaluated exactly two separate responses to the same health-related case example: one written by a human physiotherapist and one generated by the publicly available LLM ChatGPT-4. The case example was derived from a real post in a publicly accessible online health forum, where individuals can seek advice from health professionals. The original response provided by the physiotherapist served as the human reference response. The LLM response was generated using the publicly accessible ChatGPT-4 (OpenAI). The original forum question was entered verbatim as a single input without additional prompt instructions; no follow-up questions or persona or role prompts were used. The forum question, both responses, and their key characteristics (including word count and formatting) are provided in Additional File 1. It should be noted that AI-generated responses are sensitive to prompt design and that typical users are not prompt experts. The decision to use the verbatim forum question without further instructions was therefore intentional, as it reflects a common real-world use pattern in which laypeople query AI systems directly. This approach may, however, limit the quality and specificity of the generated response compared to outputs produced with optimized prompting strategies. The AI-generated response comprised approximately 290 words, was organized under three numbered sections with eight bullet points, and included detailed exercise descriptions with individual instructions. The physiotherapist’s response comprised approximately 55 words, consisted of two short paragraphs without structural headings, and provided general advice recommending a back care program without specific exercises. The AI-generated response was therefore substantially longer and more elaborately formatted than the human response. The model output was recorded as returned. The study investigated how these responses were evaluated with regard to empathy, comprehensibility, perceived discrimination, and professional quality. Data were collected in Germany using a standardized, self-administered online questionnaire.

### Sample

A total of 224 participants completed the online survey. Participants were recruited using a convenience sampling approach through open online dissemination. Eligibility criteria included being at least 18 years old and having sufficient German language proficiency to complete the questionnaire. Individuals without internet access or sufficient German proficiency were not eligible to participate. Participation was voluntary and anonymous. Because of the open recruitment strategy, a response rate could not be calculated. Given the convenience sampling approach, selection bias cannot be excluded. No formal sample size calculation was performed; the study was designed as an exploratory survey.

### Data collection

Data were collected between 26 January and 23 April 2025, using the online survey platform QUAMP^®^. The survey link was distributed through open online dissemination via multiple channels, including websites, social media platforms, professional and personal networks, and printed flyers. Before starting the survey, participants provided electronic informed consent after receiving study information. Both responses were presented in a fixed order, with the physiotherapist’s response shown first and the ChatGPT-4-generated response shown second. No randomization or counterbalancing of the presentation order was applied.

### Questionnaire

The survey instrument was developed based on a real case example from an online health forum. The case received responses from both a doctoral-level physiotherapist and a publicly available LLM (ChatGPT-4). Participants were asked to evaluate these responses across three dimensions: empathy (5 items), comprehensibility (7 items), and perceived discrimination (1 global screening item). In addition, clinical quality was assessed by experts. The primary outcomes were perceived empathy and comprehensibility; discrimination and expert-rated clinical quality were assessed as secondary outcomes. All items were rated on five-point Likert scales ranging from 1 (“absolutely not”) to 5 (“absolutely”).

For example, empathy was assessed using items such as: “Does the response demonstrate empathy?” Comprehensibility was evaluated using items such as: “Is the response easy to understand?” Perceived discrimination was assessed with the item: “Does the response contain any discriminatory content?”

Additionally, the professional quality of the responses was evaluated by three practicing physiotherapists using a separate online questionnaire focusing on clinical accuracy, medical terminology, comprehensiveness. These expert assessments were conducted independently. Given the exploratory nature of this assessment, a small expert sample was considered appropriate. The survey items were developed deductively based on established dimensions of healthcare communication quality – empathy, comprehensibility, and non-discrimination – as identified in the existing literature [14–19]. The questionnaire was subsequently refined through pretesting with a small convenience sample to enhance clarity and relevance.

### Data analysis

Data analysis was performed using IBM SPSS Statistics (Version 29). Descriptive statistics, including means, standard deviations, and frequencies, were calculated. Depending on the scale level and distribution of the data, Pearson or Spearman correlation analyses were conducted to examine relationships between variables. Correlation coefficients were interpreted using absolute values (|r| or |rho|) as follows: values of 0.00–0.29 indicated weak correlations, 0.30–0.59 moderate correlations, and ≥ 0.60 strong correlations. Additionally, binary logistic regression analyses were performed to examine whether participant characteristics were associated with overall LLM dominance in empathy and comprehensibility ratings. For these analyses, dimension-level dominance variables were dichotomized into LLM dominant versus not LLM dominant (human dominant and equal combined). Predictor variables included age (continuous), sex (female/male), native German language status (yes/no), highest educational attainment (ordinal), and having a health-related professional background (yes/no). Because of the small number of participants reporting diverse sex (*n* = 3), regression analyses were restricted to female and male participants. Odds ratios (ORs) with 95% confidence intervals (CIs) were reported. Given the high prevalence of LLM dominance, regression results were interpreted cautiously. To compare paired ordinal ratings between the human response and the LLM response, Wilcoxon signed-rank tests were performed. A post-hoc sensitivity analysis using G*Power 3.1 [20] indicated that with a sample size of 224 paired observations, a two-tailed significance level of 0.05, and 80% statistical power, the Wilcoxon signed-rank test was able to detect a minimum effect size of Cohen’s d = 0.17, corresponding to a small effect. Statistical significance was defined as *p* < .05. Missing data were not imputed; analyses were conducted using available data. As a sensitivity analysis, all Wilcoxon signed-rank tests were repeated after excluding participants who reported diverse sex (*n* = 3) to assess the robustness of the findings. Results remained unchanged in direction, magnitude, and significance across all comparisons. Due to the anonymous design of the online survey and the open dissemination strategy, data on the number of individuals who accessed the survey link or started but did not complete the questionnaire were not available. A participant flow diagram and a non-response analysis could therefore not be provided.

## Results

The AI-generated response was substantially longer and more elaborately structured than the human response (approximately 290 vs. 55 words), which must be considered when interpreting the expert ratings. Three physiotherapists independently rated both responses across three items assessing clinical accuracy (Median: human = 2 (middle), LLM = 3 (high)), use of medical terminology (Median: human = 3 (partially), LLM = 4 (rather better)), and comprehensiveness of content (Median: human = 2 (rather not), LLM = 5 (high)). Inter-rater reliability was acceptable, with an average-measures ICC of 0.826, *p* = .009, indicating good agreement among raters.

The final analytical sample comprised 224 participants. Their mean age was 37.0 years (standard deviation, 15.8). Most participants were native German speakers (90.2%), while 9.4% reported a different first language. Regarding educational attainment, nearly half of the sample held a university degree (48.2%). Overall, 37.1% of participants had a degree in a health-related field (Table 1: Sample Characteristics).

**Table 1:** Sample characteristics

	*n* = 224
Age; mean (sd)	37.0 (15.8)
**Sex**; n (%)	
female	154 (68.8)
male	67 (29.9)
divers	3 (1.3)
**Native German Speaker**; n (%)*	
yes	202 (90.2)
no	21 (9.4)
**Highest educational attainment**; n (%)	
Lower secondary school certificate	3 (1.3)
Intermediate secondary school certificate	10 (4.5)
University entrance qualification	45 (20.1)
Vocational training	58 (25.9)
University degree	108 (48.2)
**Degree in a health-related field**; n (%)*	
yes	83 (37.1)
no	140 (62.5)

*Percentages may not sum to 100% due to missing values

Overall, 71.9% of participants did not rate the human response as superior on any item, whereas only 6.3% did not rate the LLM-generated response as superior on at least one item. Ratings indicating equal evaluations were broadly distributed across participants, suggesting differentiated rather than polarized evaluation patterns. Across all evaluated items, 88.8% of participants tended to rate LLM-generated responses as superior to human responses, whereas 11.2% showed no such tendency (Table 2). All Cronbach’s alpha (> 0.709) showed sufficient to high internal consistencies.

**Table 2:** Comparison between human and AI-counseling

	*N* = 224					Wilcoxon signed rank Z, *p*-value
	*n* (%)*			Median		
Construct	Human better	Equal	LLM better	Human	LLM	rank Z, p-value
**Empathy**						
To what extent is the response formulated in a respectful manner?	6 (2.7)	41 (18.3)	164 (73.2)	3	5	**-11.152**,** < 0.001**
To what extent does the response consider the patient’s needs?	6 (2.7)	28 (12.5)	177 (79.0)	2	5	**-11.544**,** < 0.001**
To what extent is the response empathetic?	6 (2.7)	28 (12.5)	175 (78.1)	2	4	**-11.229**,** < 0.001**
To what extent does the response demonstrate an understanding of the patient’s concerns and fears?	13 (5.8)	41 (18.3)	153 (68.3)	3	4	**-10.338**,** < 0.001**
To what extent does the response demonstrate the ability to put itself in the patient’s situation?	13 (5.8)	38 (17.0)	157 (70.1)	2	4	**-10.398**,** < 0.001**
Total Empathy	12 (5.4)	20 (8.9)	192 (85.7)	2.6	4.4	**-12.091**,** < 0.001**
**Comprehensibility**						
How understandable is the information provided?	16 (7.1)	61 (27.2)	135 (60.3)	4	5	**-9.242**,** < 0.001**
How understandable is the language used (word choice and sentence structure)?	18 (8.0)	96 (42.9)	99 (44.2)	4	5	**-7.430**,** < 0.001**
Were technical terms or complex expressions explained sufficiently?	16 (7.1)	49 (21.9)	96 (42.9)	4	4	**-7.748**,** < 0.001**
How easy is it to follow and comprehend the information?	18 (8.0)	44 (19.6)	148 (66.1)	4	5	**-9.920**,** < 0.001**
How appropriate is the length of the response?	4 (1.8)	14 (6.3)	194 (86.6)	2	4	**-12.268**,** < 0.001**
How well is the response structured and organized?	2 (0.9)	31 (13.8)	174 (77.7)	2	5	**-11.541**,** < 0.001**
Would you know what your next steps should be after reading the response?	16 (7.1)	47 (21.0)	142 (63.4)	3	4	**-9.917**,** < 0.001**
Total Comprehensibility	11 (4.9)	19 (8.5)	194 (86.6)	3.1	4.4	**-12.189**,** < 0.001**
**Perceived discrimination**						
Do you perceive any wording or content in the response as discriminatory?	2 (0.9)	188 (83.9)	8 (3.6)	2	2	-1.897, 0.058

* Percentages may not sum to 100% due to missing values, bold indicates significance at 0.05 (All reported p-values are two-tailed.)

### Empathy

At the dimension level, empathy showed highly consistent patterns (Table 2). Most participants tended to rate LLM-generated responses as superior more often than the human response, whereas only a small proportion showed the opposite tendency or no clear dominance. A binary logistic regression analysis was conducted to examine whether participant characteristics were associated with overall LLM dominance in empathy ratings. The overall model was statistically significant (χ² = 16.65, *p* = .011), explaining a small proportion of variance (Nagelkerke R² = 0.13). Higher educational attainment was significantly associated with higher odds of showing LLM dominance (OR = 1.86, 95% CI [1.28–2.70], *p* = .001). Age was also significantly associated with LLM dominance, with increasing age being linked to slightly lower odds of LLM dominance (OR = 0.97, 95% CI [0.95–1.00], *p* = .028). Sex, native German language status, and having a health-related professional background were not significantly associated with LLM dominance. Given the high proportion of participants showing LLM dominance, coefficient estimates should be interpreted with caution.

### Comprehensibility

Comprehensibility showed similarly consistent patterns (Table 2). Preference distributions were highly comparable between empathy and comprehensibility. A second binary logistic regression model examined associations between participant characteristics and LLM dominance in comprehensibility ratings. The overall model was statistically significant (χ² [6] = 13.00, *p* = .043), explaining a small proportion of variance (Nagelkerke R² = 0.11). Increasing age was significantly associated with lower odds of LLM dominance (OR = 0.97, 95% CI [0.94–0.99], *p* = .003). Educational attainment, sex, native German language status, and having a health-related professional background were not significantly associated with LLM dominance. As in the previous model, the high proportion of participants showing LLM dominance warrants cautious interpretation of the estimates.

### Perceived discrimination

For discrimination, assessed using a single global screening item, evaluations were predominantly neutral. Most participants rated both responses as equal, and only small proportions indicated greater discrimination in either response. For the human response, perceived discriminatory elements were, in isolated cases, attributed to age-related assumptions (*n* = 2) and gender-related wording (*n* = 2). Free-text comments mainly referred to generalizing or potentially stereotypical statements regarding physical capacity or gender role expectations.

For the ChatGPT response, isolated perceptions of potentially discriminatory content were reported regarding age (*n* = 1), gender (*n* = 1), and origin or cultural background (*n* = 1). These perceptions primarily reflected concerns about overgeneralization or overly broad statements rather than explicitly discriminatory intent.

## Discussion

This study examined how AI-generated and human responses to the same real-world health query are perceived across key communication dimensions. Accordingly, the primary contribution of the study lies in evaluating user-perceived communication quality rather than objective clinical performance. The findings indicate a consistent preference for the AI-generated response, particularly with regard to empathy and comprehensibility, while perceptions of discrimination were largely comparable. Before attributing the more favorable comprehensibility and empathy ratings to the communicative quality of the AI-generated response, a structural confound must be acknowledged. As described in the Methods section, the AI-generated response was substantially longer and more elaborately structured than the human response, a structural confound that must be acknowledged before interpreting the results. The item showing the largest effect is appropriateness of length (Z = − 12.27), which is particularly susceptible to this confound, as participants may have associated greater length, visible structure, and content detail with higher quality independently of actual communicative skill. Whether the observed preferences reflect genuine communicative superiority of the AI-generated response or are partially attributable to these structural differences cannot be determined from the present data. Future studies should therefore ensure greater structural and content comparability between compared responses. Taken together, the results suggest that participants perceived the AI-generated response as meeting central user expectations regarding communication quality in this text-based health information scenario. These findings should not be interpreted as evidence of the broader clinical validity or appropriateness of AI-generated health advice.

### Interpretation of principal findings

One of the most notable findings is the strong preference for the AI-generated response in terms of comprehensibility. Participants frequently rated the language as clearer, the structure as more coherent, and the explanations of technical terms as more accessible. These observations align with domain-specific evaluations showing that ChatGPT can present complex information in an understandable manner [10]. Given that comprehensibility is a core prerequisite for informed health decision-making, this result is particularly relevant in light of the growing reliance on digital information sources within the general population.

A descriptive pattern further suggested that participants without German as a first language tended to perceive the AI response as more understandable. Although this observation should be interpreted cautiously because of the small subgroup size, it may point to a potential advantage of AI-generated language in reducing linguistic barriers. At the same time, prior research has highlighted the risk that digital formats may disadvantage already excluded populations [6]. Whether AI-supported communication mitigates or reproduces such disparities remains an open question that requires further investigation.

The results related to empathy are similarly noteworthy. Across all subdimensions, the AI-generated response was perceived as more empathetic. This finding is consistent with previous research reporting higher empathy ratings for chatbot responses in text-based settings [8, 9, 21]. One possible explanation is that LLMs are optimized for supportive and validation-oriented language patterns, which may systematically enhance the perception of empathy even in the absence of genuine emotional understanding. These findings highlight a distinction between perceived and experienced empathy that warrants closer conceptual attention in future research.

By contrast, perceptions of discriminatory content were rare for both responses and did not differ significantly. While this may suggest that both human and AI-generated communication can meet baseline expectations of respectful language, existing work has emphasized the importance of systematically examining bias and potential equity harms in LLM outputs [13]. Although reports of potentially discriminatory content were rare and did not differ significantly between response types, these findings should be interpreted cautiously given the reliance on a single global screening item and the very small number of observed events. The present data therefore do not allow conclusions regarding the absence of bias but instead indicate that discriminatory elements were not salient in this specific case scenario. From a safety and equity perspective, this underscores the importance of systematically evaluating both human and AI-generated health communication for subtle bias and unintended stereotyping. Future research should apply multidimensional and validated discrimination or bias assessment frameworks and include multiple case scenarios to enable more robust and generalizable conclusions.

Expert evaluations additionally favored the AI-generated response in terms of clinical accuracy and completeness. However, these findings should be interpreted with considerable caution, as the expert assessment was exploratory, based on only three physiotherapists, and limited to a single case scenario. Consequently, the present study does not establish the broader clinical appropriateness, safety, completeness, or guideline concordance of AI-generated responses. Rather than challenging existing concerns, these findings provide only preliminary observations that warrant confirmation through larger expert panels, multiple clinical scenarios, and standardized assessments of clinical quality. Beyond individual dimensions, the broadly distributed equal ratings suggest that participants engaged in differentiated evaluations rather than uniformly favoring one response type. This pattern supports the interpretation that user perceptions of AI-supported communication are nuanced and context-sensitive, echoing prior work describing the situational acceptance of digital consultation formats [4, 5].

The post-hoc sensitivity analysis indicated that the study was powered to detect effect sizes as small as Cohen’s d = 0.17. Given that all paired comparisons for empathy and comprehensibility reached significance at *p* < .001, the observed effects substantially exceeded this detection threshold, supporting the robustness of the reported preference patterns.

### Implications for digital health communication

The present findings suggest that AI-generated responses may be perceived positively in text-based informational settings. However, the study did not evaluate whether such responses are clinically appropriate, safe, or effective in real-world healthcare consultation. These aspects require further investigation before implications for clinical implementation can be drawn. Features such as consistent structure, accessible language, and supportive phrasing appear to align with user expectations in text-based environments. In health care systems facing increasing demand and resource constraints, these characteristics may help expand access to understandable health information.

At the same time, the results should not be interpreted as supporting a substitution of professional consultation. Prior research has shown that acceptance of digital counseling decreases with increasing complexity and uncertainty [4, 5], and concerns regarding inconsistent quality and potentially harmful recommendations remain relevant [12, 22]. From this perspective, AI-supported tools may be most appropriately positioned within a stepped communication model in which automated information complements, but does not replace, professional expertise.

The growing presence of AI in health information seeking also raises questions about professional roles and competencies. Ensuring the safe integration of such tools will likely require not only technical oversight but also strengthened digital competencies among health care professionals. Educational approaches that enable critical appraisal of AI-generated content may therefore become increasingly important as digital counseling formats continue to expand [23].

### Limitations

Several limitations should be considered when interpreting the findings. First, the comparison was based on a single case example and one human response, which represents a limitation of the present study. The results cannot be generalized beyond this specific case, as both response length, quality and user evaluations may vary substantially depending on the health topic, the complexity of the query, and the individual communication style of the responding professional. Future studies should include multiple case scenarios across different clinical domains to enable more robust and generalizable conclusions. Because evaluations were based on self-reported perceptions, reporting bias cannot be excluded. Although the study was sufficiently powered to detect small effects (Cohen’s d = 0.17), the ability to detect such small differences also implies that statistically significant results may not necessarily reflect practically meaningful distinctions in all cases. The clinical or communicative relevance of the observed differences should therefore be assessed independently of statistical significance.

Second, the study relied on a convenience sample recruited through open online dissemination. Although this approach enabled broad participation, selection bias cannot be excluded, and the relatively high educational attainment within the sample may limit generalizability.

Third, participants evaluated static written responses without the opportunity for interaction. Health communication is typically dynamic and allows for clarification, follow-up questions, and adaptation to individual needs. The extent to which the observed preferences would persist in interactive settings remains uncertain.

Fourth, expert assessments were conducted with a small sample and should be interpreted as exploratory. Larger expert panels and standardized evaluation frameworks would strengthen future comparisons of clinical quality.

Fifth, the two responses were presented in a fixed order without randomization or counterbalancing. Since the longer and more structured AI-generated response was presented second, any contrast effects would more likely have operated against rather than in favor of the AI response. Nevertheless, differences in lengths and formatting characteristics of both responses are therefore reported in Additional File 1. Future studies should employ counterbalanced designs and ensure greater comparability between response formats.

Finally, AI-generated responses are inherently sensitive to prompt design, model configuration, and ongoing technological development. The findings should therefore be understood as reflecting the capabilities of the model at the time of data collection rather than representing a fixed performance estimate.

### Future research

Further studies should examine multiple case scenarios across different clinical domains and levels of complexity to better understand where AI-supported communication is perceived as beneficial and where its limitations become more apparent. Research incorporating interactive dialogue formats may provide additional insight into how users engage with AI-generated advice in more realistic communication settings.

In addition, qualitative approaches could help clarify which linguistic and structural features shape perceptions of empathy and comprehensibility. Establishing robust evaluation criteria for AI-supported health communication remains an important step toward ensuring both quality and safety as these technologies become more widely integrated into digital care environments.

## Conclusion

The present study suggests that AI-generated responses can meet central user expectations regarding clarity, structure, and perceived empathy in text-based health communication. At the same time, the findings highlight that perceived communicative strength should be considered alongside ongoing concerns related to clinical appropriateness, safety, and equity.

Rather than informing recommendations regarding the implementation of AI in healthcare consultation, the present findings primarily contribute evidence on user-perceived communication quality. Future studies should determine whether these positive perceptions are accompanied by adequate clinical validity, safety, and guideline concordance.

As AI-tools become more widely used in health information seeking, their responsible integration into existing care structures will require ongoing empirical evaluation, clear quality standards, and strengthened digital competencies among both users and health care professionals. Only through such a differentiated approach can the potential of AI-supported communication be harnessed while safeguarding the quality of health care delivery.

## Supplementary Information

Below is the link to the electronic supplementary material.

Supplementary Material 1

## Data Availability

The datasets used and analyzed during the current study are available from the corresponding author upon reasonable request.

## Abbreviations

AI: Artificial intelligence
CI: Confidence interval
ICN: International Council of Nurses
ICMJE: International Committee of Medical Journal Editors
LLM: Large language model
OR: Odds ratio
SD: Standard deviation
SPSS: Statistical Package for the Social Sciences
