# Supplementary material for: User evaluation of AI- and human-generated responses in digital health communication: a paired survey study
Source: BMC Health Serv Res. 2026 Aug 19;26:1155. doi: 10.1186/s12913-026-15305-4 (PMC13488572; doi:10.1186/s12913-026-15305-4)
Supplement: Supplementary file 1 [file 12913_2026_15305_MOESM1_ESM.docx]

**Original Health Forum Query:**

Hello dear forum!

A little about me: I am female, 31 years old, 157 cm tall, and weigh 60 kg. I have a desk job, but I do try to keep my daily routine reasonably active. I walk to and from work, always go for a walk at lunchtime (about one hour in total), walk the dog every day, and also exercise regularly (currently bodyweight exercises, strength training, and swimming).

My problem is the following: When I sit leaning forward for a longer period, or work in a bent-over position, for example in the garden, I have difficulty straightening up again afterwards. I then feel a very strong pulling sensation in my lower back and have to remain slightly bent forward for a while (perhaps about 1 minute) before I can stand fully upright again.

Which stretching and/or strengthening exercises might help me with this?

Thank you in advance!

[Originally posted in German on a publicly accessible online health forum. Translated by the authors.]

**ChatGPT-4-Generated Response**

Hello and welcome!

It sounds as though you are dealing with tension or perhaps a slight postural misalignment in your lower back. The symptoms you describe could result from a combination of muscular tension and a certain imbalance in the trunk muscles, particularly around the spine and the hip musculature.

Here are a few exercises that might help you:

1. Stretching exercises for the lower back:

*Cat-Cow Stretch:* This yoga exercise helps promote spinal mobility and stretch the lower back. Start on all fours, arch your back downward while inhaling (cow position), and round your back while exhaling (cat position). Make sure to move your head gently along with the movement.

*Child's Pose:* Sit on your heels, bend forward, and gently place your forehead on the floor. Extend your arms forward to intensify the stretch. This exercise stretches the lower back muscles and helps relieve tension.

*Supine Twist:* Lie on your back, place your feet flat on the floor, and let your knees drop to one side while rotating your upper body in the opposite direction. This helps mobilize the spine and relax the lower back.

2. Strengthening exercises for the lower back and core:

*Glute Bridge:* Lie on your back, place your feet on the floor, and lift your pelvis by engaging your gluteal muscles. This exercise strengthens the lower back, the gluteal muscles, and the hamstrings, which helps stabilize posture.

*Plank (Forearm Support):* This exercise strengthens your core, which helps stabilize the spine and prevent tension. Make sure to keep your back straight during the exercise to engage the correct muscles.

*Superman:* Lie face down on the floor and simultaneously lift your arms and legs so that your body assumes a "Superman" position. This exercise strengthens the lower back and the posterior chain muscles.

3. Postural corrections in daily life:

*Upright Sitting:* Make sure to maintain an upright posture when sitting. If possible, use a chair that provides slight support for the lower back. Sit up straight with your shoulders pulled back and your head aligned with the spine.

*Stretching Breaks:* If you sit for extended periods, try to stand up regularly and stretch or take a short walk. This helps prevent tension in the lower back.

If the symptoms persist or worsen, it would be advisable to consult a physiotherapist or orthopedic specialist for a more precise diagnosis and individualized treatment.

Hopefully these exercises and tips will be of help to you!

*[Originally generated by ChatGPT-4 (OpenAI) in March 2024 in response to the verbatim forum query. No prompt modifications, follow-up questions, or role instructions were applied. The output was recorded as returned. Translated by the authors.]*

**Physiotherapist Response**

Hello,

Sitting or working in a bent-forward position for extended periods is not recommended, as this places considerable strain on the back — as you have already experienced yourself. Many postural errors can be avoided. Relearning new movement patterns takes some time, but it can be learned. Back schools can help with this. Ask your health insurance provider where such a back school is offered. Many physiotherapy practices offer these back schools.

Kind regards

*[Original response provided by a professional physiotherapist in the publicly accessible online health forum. Translated by the authors.]*
